# Development of One-Step Tetra-primer ARMS-PCR for Simultaneous Detection of the *Angiotensin Converting Enzyme (ACE)* I/D and *rs4343* Gene Polymorphisms and the Correlation with CAD Patients

**Published:** 2019

**Authors:** Mohammad Mehdi Heidari, Mehdi Hadadzadeh, Hossein Fallahzadeh

**Affiliations:** 1. Department of Biology, Faculty of Science, Yazd University, Yazd, Iran; 2. Department of Cardiac Surgery, Afshar Hospital, Shahid Sadoughi University of Medical Sciences, Yazd, Iran; 3. Research Center of Prevention and Epidemiology of Non-Communicable Disease, Shahid Sadoughi University of Medical Sciences, Yazd, Iran

**Keywords:** Angiotensin converting enzyme (*ACE*) gene, Coronary artery disease, Insertion/Deletion polymorphism

## Abstract

**Background:**

The *Angiotensin Converting Enzyme (ACE)* Insertion/Deletion and *rs-4343* gene polymorphisms could be associated with pathogenesis of essential hypertension and cardiovascular disorders and Coronary Artery Disease (CAD). In the present study, a fast and novel approach of multiplex Tetra-Primer Amplification Refractory Mutation System-PCR (T-ARMS-PCR) was developed for simultaneous detection of two SNPs including *ACE I/D* (rs4340) and 2350A>G (rs4343) of *Angiotensin Converting Enzyme* (*ACE*) gene.

**Methods:**

The present research was performed using 148 blood samples taken from patients with CAD and 135 healthy individuals. One set of inner primers (for rs4343) and one set of outer primer pairs were designed for genotyping of Insertion/Deletion and rs4343 polymorphisms in single tube T-ARMS-PCR.

**Results:**

Our results manifested that genotypes and alleles frequency of the *ACE* polymorphisms showed no statistically significant association between CAD patients and the control group. In addition, complete concordance was seen between sensitive Tetra-ARMS-PCR and sequencing method.

**Conclusion:**

The technique is the first work for simultaneous detection of Insertion/Deletion polymorphism and rs4343 SNPs in *ACE* gene and the results were entirely according to those from an independent procedure.

## Introduction

Coronary Artery Disease (CAD) is the result of atherosclerotic occlusion of the coronary arteries. Most of all deaths in the developing world are due to Cardiovascular Disease such as hypertension and the diseases caused by atherosclerosis 1. Various genetic variations are known to affect coronary atherosclerotic plaques 2–4.

A key physiological regulator of blood pressure is the Renin-Angiotensin System (RAS). An enzyme which determined the vasoactive peptide Angiotensin-II in RAS pathway is angiotensin converting enzyme (ACE or Peptidyl-Dipeptidase A). The most common polymorphism in *ACE* gene is a 287-base-pair Insertion/Deletion (I/D) *Alu* element within the intron 16 of gene (rs4340 SNP). It was found that this polymorphism is related to the ACE activity level which is drastically reduced in homozygous deletion carriers (DD) when compared to homozygous II carries, while heterozygous ID carriers show middle activity level 5. Another single nucleotide polymorphism in *ACE* gene is a silent and synonymous coding polymorphism 2350A>G (rs-4343) in exon 17. The association between ACE I/D (rs4340) and 2350A>G (rs4343) polymorphisms has been found in several studies including high blood pressure, systemic lupus erythematous, CAD, diabetic nephropathy, Alzheimer’s disease and renal diseases 6,7.

In this study, a case-control evaluation of ACE polymorphisms was done to examine their putative association with CAD in an Iranian population. Since the distance between the *ACE* I/D (rs4340) and 2350A>G (rs4343) polymorphisms is 139 *bp*, a new Tetra primer-amplification refractory mutation system-PCR (T-AR MS-PCR) method was developed for simultaneous detection of these SNPs in single reaction.

## Materials and Methods

### Patients

The present study was a case-control association study. Peripheral blood samples were collected from 283 subjects (148 CAD patients and 135 age and sex matched healthy controls with no family history of CAD) who referred to cardiac centers in Afshar Hospital (Yazd, Iran) from 2012 to 2015. CAD patients were selected according to the coronary angiography and Electrocardiogram (ECG) criteria 8. Routine biochemical measurements were obtained from patients with CAD and healthy subjects including determination of fasting lipid levels High-Density Lipoprotein (HDL), triglycerides, and Low-Density Lipoprotein (LDL) levels (Table 1).

**Table 1.** The summary of the clinical characteristics of coronary atherosclerosis patients and controls

	**Patients (n=148)**	**Controls (n=135)**
**Male gender (%)**	73	69
**Age, years**	52.6±7.2	51.9± 6.7
**Smokers (%)**	26	22
Body mass index (*kg/m*^2^) ^*^	26.5±2.6	25.3±2.1
Cholesterol, mg/dl^*^	208.6±53.4	182.3±46.4
**LDL-C, *mg/dl***	125.4±44.8	112.9±43.9
**HDL-C, *mg/dl***	42.8±8.9	48.7±12.5
TGs, mg/dl^*^	201.2±108.2	149.3±92.5
Systolic blood pressure (*mmHg*) ^*^	153.2±0.6	116.9±0.3
Diastolic blood pressure (*mmHg*) ^*^	97.5±0.4	78.3±0.4

* p-value <0.05

The control group consisted of 135 unrelated healthy age, sex and ethnicity matched subjects with normal or near-normal angiography results. Our protocol was approved by Yazd University Human Research Ethics Committee, in accordance with the revised declaration of Helsinki and all participants gave their informed consents for the molecular analysis. Genomic DNA was extracted from the whole blood by a salting out method.

### Genotyping

All primers used in this research were designed by a web primer design program, Primer1, accessible at http://primer1.soton.ac.uk/primer1.html (Table 2). The PCR reaction was carried out in a total volume of 25 *μl*, containing 50 *ng* of genomic DNA as template, 5 *pmol* of each outer primer, 10 *pmol* of each inner primer, and 1X multiplex PCR master mix (Yekta Tajhiz Azma Co., Tehran, Iran). PCR amplification (Touchdown) was performed with an initial denaturation at 95 °*C* for 2 *min*, followed by denaturation at 95 °*C* for 20 *s*, first annealing at 69 °*C* to 60 °*C* (10 cycles) and the remaining cycles (25 cycles) were carried out with annealing at 60 °*C* for 1 *min* and extension at 72 °*C* for 1 *min*, followed by a final extension for 5 *min*. The amplified products were run on non-denaturing polyacrylamide gel electrophoresis (6%) and were stained with silver staining method.

**Figure 1.**
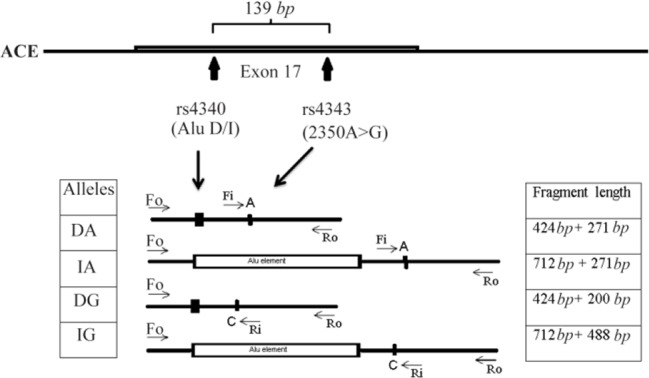
Schematic illustration of primer interactions for *ACE* Multiplex T-ARMS-PCR assay. Fo and Ro are outer primers acting as control primers, and Fi and Ri are inner primers for 2350 nucleotide substitution (rs4343).

The *ACE* I/D (rs4340) and 2350A>G (rs4343) polymorphisms are located in intron 16 and exon 17, respectively, with 139 *bp* distance. Four primers were used in the same PCR. Two primers, Fo and Ro were designed to amplify a 712 *bp* band (I allele) and 424 *bp* band (D allele) which served as control bands for the success of the amplification (Figure 1). Two specific primers, Fi and Ri for rs4343 with complementary 3'-end nucleotide to corresponding polymorphisms, were introduced (Table 2).

**Table 2.** PCR primer sequences

**ACE gene**		**Primer sequence**	**Amplicon size**
**(SNP ID: rs4343, A2350G)**			
	Forward inner primer A allele	5'-TCTGACGAATGTGATGGCCCCA-3'	271 *bp*
	Reverse inner primer G allele	5'-AACAGGTCTTCATATTTCCGGTAC-3'	200 *bp* with I allele 488 *bp* with D allele
**Control fragment (SNP ID: rs4340, I/D)**			
	Forward outer primer	5'-GAGGCTGAGATGGAAGGATTG-3'	712 *bp* for I allele
	Reverse outer primer	5'-GCTCTCCCAACACCACATTAC-3'	424 *bp* for D allele

### Statistical analysis

The Chi-square goodness-of-fit test was used to investigate the association between the two groups. Values of p<0.05 were considered statistically significant. Statistical package for the social sciences (SPSS) software (IBM SPSS 22, SPSS Inc., Chicago, IL., USA) was used to perform the statistical analysis.

## Results

The average serum levels of cholesterol and triglycerides were comparable between cases and controls, whereas their levels were higher in the CAD patients (p<0.05). However, no significance differences were observed in the LDL and HDL levels between both groups (Table 1). Both of means in systolic and diastolic blood pressure showed important statistical differences in patients and controls (p<0.05).

**Figure 2.**
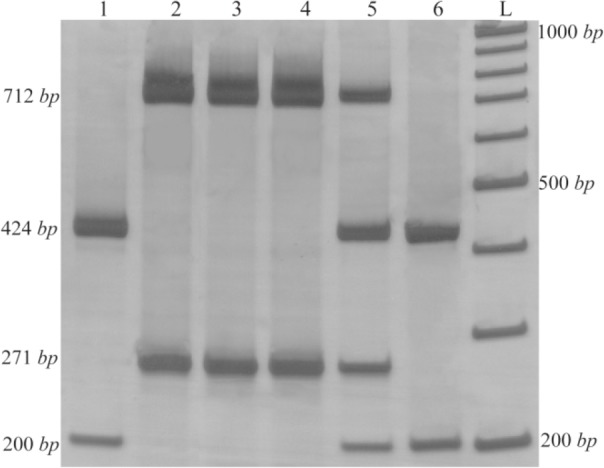
Results of T-ARMS-PCR of *ACE* polymorphisms (rs4043 and rs4343). Lanes 1 and 6: DDGG genotype, lanes 2, 3 and 4: IIAA genotype and lane 5: DIAG (IA/DG) genotype.

The simple Tetra-ARMS-PCR method can determine the arrangement of alleles on chromosome. This arrangement means that insertion allele (ACE I allele) and A allele (in rs4343) are found on one chromosome (IA), which is referred to as the coupling, or Cis configuration. Alternatively, one chromosome might bear the deletion allele (ACE D allele) and A allele (DA) which is called the repulsion, or Trans configuration. The identification of each genotype arrangement (Cis or Trans state) was performed by comparing to the expected fragment lengths (Table 3). PCR products with these allele arrangements including IA arrangement were: 712 and 271 *bp* fragments, IG arrangement: 712 and 488 *bp* fragments, DA arrangement: 712 and 271 *bp* fragments and DG alleles: 424 and 200 *bp* fragments (Figure 2).

**Table 3.** Amplification patterns observed and those expected according to ACE polymorphisms genotypes

**Genotype**	**DDAA**	**DDAG**	**DDGG**	**DIAA**	**DIAG**		**DIGG**	**IIAA**	**IIAG**	**IIGG**

**Cis/Trans**	DA/DA	DA/DG	DG/DG	DA/IA	IA/DG	IG/DA	IG/DG	IA/IA	IA/IG	IG/IG
**Amplicon (*bp*)**	-	-	-	712 *bp*	712 *bp*	712 *bp*	712 *bp*	712 *bp*	712 *bp*	712 *bp*
	-	-	-	-	-	488 *bp*	488 *bp*	-	488 *bp*	488 *bp*
	424 *bp*	424 *bp*	424 *bp*	424 *bp*	424 *bp*	424 *bp*	424 *bp*	-	-	-
	271 *bp*	271 *bp*	-	271 *bp*	271 *bp*	271 *bp*	-	271 *bp*	271 *bp*	-
	-	200 *bp*	200 *bp*	-	200 *bp*	-	200 *bp*	-	-	-

D and I: Deletion/Insertion allele (rs4340 SNP); A and G: wild type and mutant allele (rs4343 SNP).

The allelic discrimination for all patients with CAD and normal samples was performed through T-ARMS-PCR by two outer primers in the presence of two inner specific primers for *ACE* polymorphisms. Furthermore, genotypes of a limited number of samples were analyzed through direct sequencing (for verification). It was found that genotyping results obtained from both methods were fully concordant. Two types of polymorphism in the *ACE* gene, ACE I/D (rs4340) and 2350A>G (rs4343) were analyzed and their genotype distributions and allele frequencies in CAD patients and healthy subjects are shown in table 4.

**Table 4.** Genotype and allele frequencies of ACE variants in patients and controls

**ACE (rs4340)**	**Patients (n=148)**	**Controls (n=135)**	**OR (95% CI)**	**p-value**
**Co-dominant model**				
DD	69 (46.6%)	73 (54.1%)	1	
DI	54(36.5 %)	47 (34.8%)	1.076 (0.661–1.751)	0.805
II	25 (16.9%)	15 (11.1%)	1.626 (0.817–3.235)	0.176
**Dominant model**				
DD	69 (46.6%)	73 (54.1%)		
DI+ II	79 (53.4%)	62 (45.9%)	0.742 (0.465–1.184)	0.235
**Recessive model**				
DD+DI	123 (83.1%)	120 (88.9%)		
II	25 (16.9%)	15 (11.1%)	0.615 (0.309–1.223)	0.176
**Overdominant**				
DD+ II	94 (63.5%)	88 (65.2%)		
DI	54 (36.5%)	47 (34.8%)	0.930 (0.571–1.513)	0.805
**Allele frequency**				
D	192 (64.9%)	193 (71.5%)		
I	104 (35.1%)	77 (28.5%)	0.737 (0.516–1.052)	0.104
***ACE* (rs4343)**				
**Codominant model**				
AA	64 (43.2%)	62 (45.9%)	1	
AG	61 (41.2%)	55 (40.7 %)	1.020 (0.635–1.639)	1.000
GG	23 (15.6%)	18 (13.4%)	1.195 (0.614–2.329)	0.617
**Dominant model**				
AA	64 (43.2%)	62 (45.9%)		
AG+GG	84 (56.8 %)	73 (54.1%)	0.897 (0.561–1.434)	0.720
**Recessive model**				
AA+AG	125 (84.4%)	117 (86.6%)		
GG	23 (15.6%)	18 (13.4%)	0.769 (0.399–1.483)	0.508
**Overdominant**				
AA+GG	87 (58.8%)	80 (59.3%)		
AG	61 (41.2%)	55 (40.7 %)	0.981 (0.610–1.576)	1.000
**Allele frequency**				
A	189 (63.8%)	179 (66.3%)		
G	107 (36.2%)	91 (33.7%)	0.898 (0.635–1.270)	0.597

OR, odds ratio; CI, Confidence interval.

All the patients and controls were genotyped for rs4340 and rs4343 polymorphisms by logistic regression analysis according to co-dominant, dominant and recessive models (p>0.05). Our result showed that there was no significant difference in *ACE* polymorphisms genotype distribution and allele frequency between CAD patients and the control group. Table 5 shows the results of the haplotype analysis.

**Table 5.** Haplotype analysis of AGT polymorphisms in CAD patients and controls

**Genotype**	**Cis or Trans**	**Patients (n=148)**	**Controls (n=135)**	**OR (95% CI)**	**p-value**
**DDAA**	DA/DA	31	35	1	
**DDAG**	DA/DG	17	18	0.844 (0.415–1.713)	0.719
**DDGG**	DG/DG	17	16	0.965 (0.467–1.996)	1.000
**DIAA**	DA/IA	18	19	0.845 (0.423–1.688)	0.725
**DIAG**					
	DA/IG	23	16	1.369 (0.689–2.717)	0.392
	DG/IA	13	14	0.832 (0.376–1.841)	0.689
**DIGG**	DG/IG	4	2	1.847 (0.333–10.251)	0.686
**IIAA**	IA/IA	15	8	1.790 (0.734–4.368)	0.276
**IIAG**	IA/IG	8	7	1.045 (0.368–2.963)	1.000
**IIGG**	IG/IG	2	0	1.014 (0.995–1.033)	0.499

OR, odds ratio; CI, Confidence interval.

## Discussion

Numerous experimental studies have suggested that ACE is a key enzyme in the production of angiotensin II in the modulation of cardiac growth. Genotyping of *ACE* gene polymorphisms has provided a genetic marker for several human heart diseases such as ischemic heart disease, coronary artery stenosis, myocardial infarction and ischemic cerebrovascular disease 9,10. Association of these markers such ACE I/D (rs4340) and 2350A>G (rs4343) polymorphisms has been found previously by researchers in different populations (Table 6). Differences in various populations are due to variations in genetic and environmental background.

**Table 6.** The results of different studies on *ACE* gene polymorphisms

**Study (ACE I/D SNP)**	**Population**	**Number**	**Genotype (%)**			**Allele (%)**		**p-value**

			**II**	**ID**	**DD**	**I**	**D**	
**Firouzabadi *et al*^7^**	Iran	183	21.31	39.89	38.79	41.25	58.74	0.092
**Poorgholi *et al*^13^**	Iran	676	14.3	38.8	46.9	33.7	66.3	0.494
**Saddick *et al*^11^**	Saudi Arabia	100	17	30	53	32	68	0.40
**Sameer *et al*^14^**	India (Kashmiri)	52	23.7	30.77	46.15	38.46	61.53	0.24
**Rigat *et al*^15^**	France	199	N.A.	N.A.	N.A.	42.7	57.3	N.A.

**Study (rs4343 SNP)**	**Population**	**Number**	**Genotype (%)**			**Allele (%)**		**p-value**

			**AA**	**AG**	**GG**	**A**	**G**	
**Firouzabadi *et al*^7^**	Iran	183	24.59	48.63	26.77	48.9	51.09	0.182
**Jia *et al*^16^**	China	717	39.9	47.6	12.6	64.08	35.91	0.084
**Abdollahi *et al*^17^**	U.K.	2753	11.11	60.69	28.18	59.62	40.31	0.12
**Ancelin *et al*^12^**	French	470	22.18	52.02	25.81	50.85	49.14	0.32
**Jiang *et al*^18^**	China	75	34.66	46.67	18.67	58.00	42.00	0.0117

The standard PCR instruments are highly desirable for scientific studies of large number of patients and for diagnostic analysis, economical and fast assays. For conventional genotyping of *ACE* gene polymorphisms, scientists developed PCR-based methods which allow discrimination of different ACE genotypes. For diagnostic analysis in large numbers of patients, fast and acute assays that can be carried out with standard PCR equipment are highly desirable.

In this research, a rapid, reliable, sensitive and easy to use assay for detecting *ACE* I/D (rs4340) and 2350 A>G (rs4343) polymorphisms in our population was developed and compared with reported results in different investigations. A one-step Tetra-primer ARMS-PCR assay was designed and genotype results were obtained that were fully concordant with direct sequencing of randomly selected samples (n=18). Furthermore, one-step Tetra-primer ARMS-PCR assay is easy to perform and needs only a small amount of standard PCR reagents and is applicable to identify the genetic variations without special equipment.

For the first time, Saddick *et al* used a novel approach of tetra primer ARMS-PCR for genotyping *ACE* I/D (rs4340) polymorphism in pregnant women with Mild Gestational Hyperglycemia (MGH) 11. In this manner, simultaneous distinction of homozygous and heterozygous samples for *ACE* I/D (rs4340) and 2350A>G (rs4343) polymorphisms was achieved by tetra primer ARMS-PCR. Our data showed that none of these polymorphisms was associated with CAD in Iranian patients. There were no statistically important associations between genotype distribution, allele and haplotype frequencies between cases and healthy controls. Our results are in agreement with some previous research such as Poorgholi *et al* and Ancelin *et al*, who did not find any association between *ACE* I/D (rs4340) and 2350A>G (rs4343) polymorphisms and CAD development 12,13. These results show that cardiovascular disorders may be caused not only by angiographic characteristics but also by combination of some genetic factors associated with environmental risk factors. Moreover, the disagreement between reported results could be not only related to difference in ethnicity and sample size, but also connected to differences in clinical presentation, severity of disorders, age and gene-environment interactions.

## Conclusion

In conclusion, it should be noted that a simultaneous detection method for two different variations in *ACE* gene was presented by one-step Tetra-primer ARMS-PCR assay. However, our study does not report a significant association of *ACE* I/D (rs4340) and 2350A>G (rs4343) polymorphisms in Iranian patients with coronary artery disease. Further researches are required to completely understand the role *ACE* variations in different populations.
